# Advances in Musculoskeletal Tissue Biology and Regenerative Techniques: Innovative Approaches to Bone Repair and Cartilage Regeneration in Orthopedic Surgery

**DOI:** 10.7759/cureus.106623

**Published:** 2026-04-07

**Authors:** Manjudev S Nakkaragundi, Abhay Harsh Kerketta, Parishekh Paulraj Jawahar, Karun Jain, Deepankar Satapathy, Aishwarya Singh

**Affiliations:** 1 Department of Orthopaedics, S Nijalingappa Medical College and HSK Hospital and Research Centre, Bagalkot, IND; 2 Department of Orthopaedics, Netaji Subhas Medical College and Hospital, Jamshedpur, IND; 3 Department of Orthopaedics, Apollo Hospitals OMR, Chennai, IND; 4 Department of Orthopaedics, Pushpanjali Medical Centre, New Delhi, IND; 5 Department of Orthopaedics, All India Institute of Medical Sciences, Bibinagar, IND; 6 Department of Pathology, Shri Guru Ram Rai Medical and Health Sciences, Dehradun, IND

**Keywords:** biomaterials, cartilage regeneration, musculoskeletal repair, orthobiologics, tissue engineering

## Abstract

Musculoskeletal disorders involving bone and cartilage remain a major clinical burden because these tissues have limited intrinsic regenerative capacity, and conventional orthopedic treatments often fail to restore durable structural and functional outcomes. Advances in musculoskeletal tissue biology have clarified the complex cellular, molecular, immunological, and biomechanical regulators of healing, forming a strong foundation for the development of next-generation regenerative therapies. Emerging strategies, including orthobiologic preparations, stem cell-based interventions, biomimetic and smart biomaterial scaffolds, nanotechnology-driven platforms, and three-dimensional bioprinting, have demonstrated improvements in matrix synthesis, angiogenesis, and inflammatory modulation in preclinical models and early clinical studies, with recent advances also showing improved control of cell differentiation, enhanced scaffold integration, and more precise tissue architecture. Clinical evidence suggests benefits, including enhanced fracture healing, improved repair of cartilage defects, and symptomatic relief in early osteoarthritis. However, outcomes remain variable due to heterogeneity in biologic preparations and study designs. Despite these advances, major challenges persist, including variability in biologic preparations, limited long-term clinical evidence, difficulties achieving vascularized and mechanically stable constructs, and evolving regulatory and manufacturing constraints. This review synthesizes contemporary mechanistic insights and critically evaluates recent regenerative approaches for bone repair and cartilage restoration, highlighting therapeutic progress, persistent limitations, and translational barriers. By identifying key knowledge gaps and outlining priority directions, this work aims to advance the development of reliable, durable, and patient-tailored regenerative solutions capable of transforming orthopedic care.

## Introduction and background

Musculoskeletal disorders represent a major global health burden and are among the leading causes of disability and reduced quality of life worldwide [1]. Among these, osteoarthritis and cartilage injuries are particularly common and are major contributors to chronic pain, functional limitation, and reduced quality of life in affected individuals. Injuries and degenerative diseases of bone and articular cartilage are among the conditions that are particularly consequential because of their limited intrinsic healing capacity [2]. Conditions such as traumatic defects, osteoarthritis, avascular necrosis, and complex fractures often fail to regenerate adequately through natural repair mechanisms [3]. This results in persistent pain, structural abnormalities, and functional impairment [3]. For example, cartilage damage in osteoarthritis or delayed fracture healing in long bones often fails to restore normal structure and function without intervention. Conventional orthopedic procedures, including autografts, allografts, microfracture, and joint arthroplasty, have contributed significantly to clinical practice but are characterized by serious limitations, such as donor-site morbidity, limited tissue availability, immune rejection, graft failure, and biomechanical mismatch with adjacent tissue [4]. These limitations highlight the need for biological approaches that not only repair defects but also restore native tissue structure and function [5].

Over the past decade, musculoskeletal tissue biology has advanced significantly [6]. Bone is now recognized as a dynamic system regulated by coordinated interactions among osteoblasts, osteoclasts, osteocytes, endothelial cells, and immune mediators [7]. Mechanical loading, microvascular networks, and inflammatory signals are vital in the regulation of osteogenesis and remodeling [8]. Parallel to this, avascular, aneural, structurally zonal articular cartilage has been a central focus of regenerative research due to its lack of self-healing upon damage [9]. Recent developments in developmental biology and mechanotransduction have clarified the signaling pathways that regulate chondrocyte phenotype, matrix remodeling, and cartilage degeneration [10]. These insights provide a stronger foundation for the design of regenerative therapies.

Rationale for regenerative approaches

Based on these biological findings, regenerative orthopedics has quickly become an interdisciplinary field, integrating orthobiologics (biologically derived materials such as platelet-rich plasma and bone marrow aspirate used to enhance healing), stem cell therapy, tissue engineering, biomaterials science, nanotechnology, and bioprinting [11]. Platelet-rich plasma, bone marrow aspirate concentrate, and next-generation marrow-derived products are orthobiologic systems that provide targeted delivery of growth factors and cytokines that regulate inflammatory and endogenous repair pathways [12]. Stem cell therapies, especially those using bone marrow mesenchymal stromal cells, adipose tissue, synovium, or periosteum, possess multipotent regenerative capacity that has been shown to affect immune regulation and matrix synthesis [13]. Cell-free methods, such as extracellular vesicles and exosomes, have been considered alternatives due to their lower immunogenicity, greater safety, and strong paracrine-based biological efficacy [14].

Meanwhile, biomaterials engineering has led to the development of scaffolds (three-dimensional structural frameworks designed to support cell growth and tissue formation) that can recapitulate the biochemical milieu and hierarchical organization of native bone and cartilage [15]. Optimization of natural polymers, synthetic polymers, bioactive ceramics, and hybrid composites with respect to mechanical strength, porosity, degradation rate, and surface chemistry has been shown to facilitate cell adhesion, proliferation, and differentiation [16]. Smart biomaterials, including mechanically adaptive, immunomodulatory, or controlled-release versions, further increase the precision of therapy [17]. Nanotechnology has also enabled breakthroughs through the ability to tune scaffold architecture at the nanoscale, targeted delivery of bioactive molecules, and enhanced control of cell-matrix interactions [18]. New functions of additive manufacturing and three-dimensional bioprinting have also enabled patient-specific defect reconstruction, osteochondral unit fabrication, and spatial organization of cells and growth factors [19]. These new technologies are paired with concepts from mechanobiology (the study of how mechanical forces influence cellular behavior), combining specific mechanical stimulation with biologic therapies to direct tissue development and maximize functional outcomes [20]. In addition, emerging approaches such as exosome-based therapies, artificial intelligence (AI)-assisted modeling, and organ-on-a-chip systems are improving the ability to predict therapeutic responses and optimize regenerative strategies [21].

**Figure 1:**
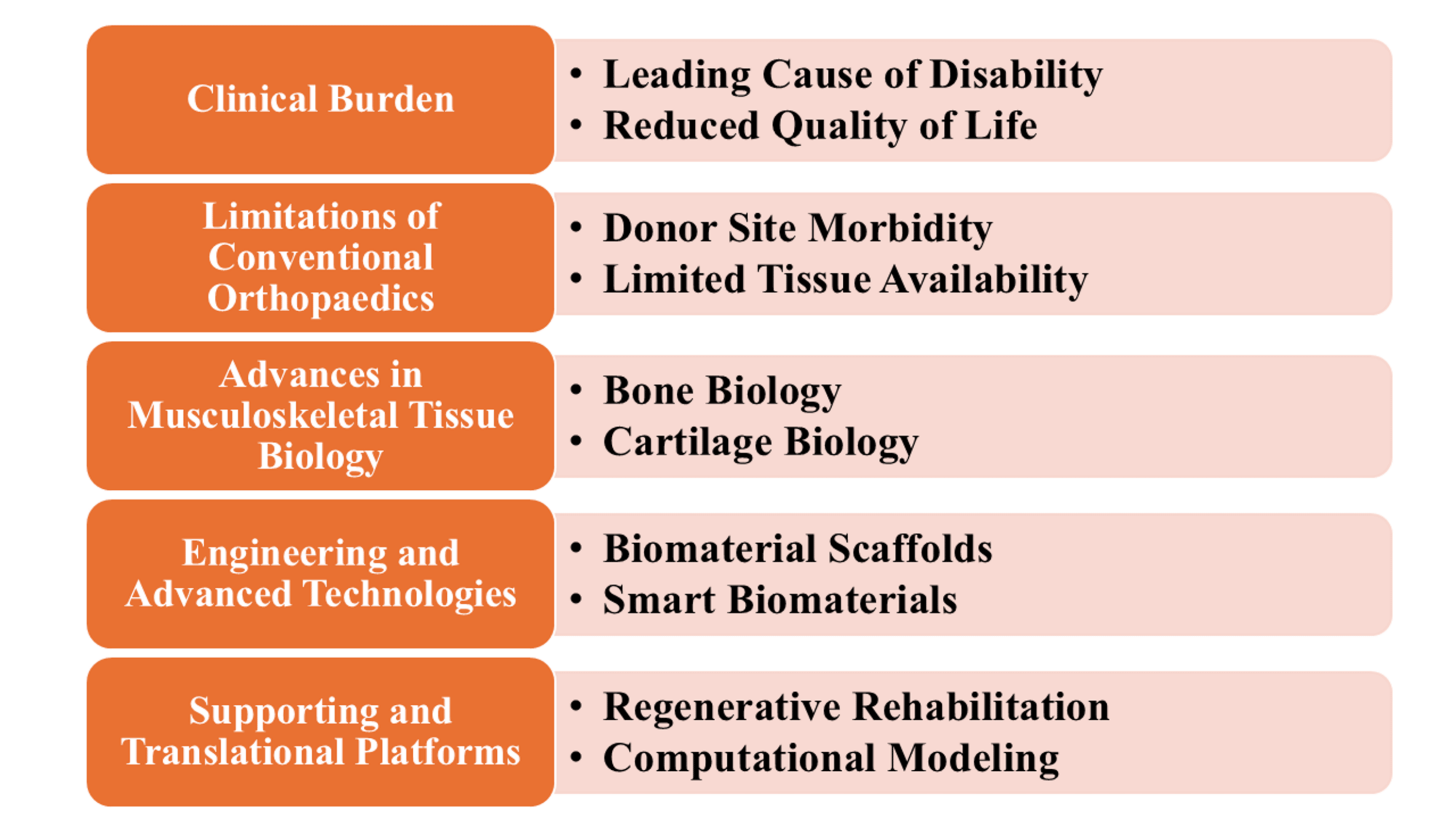
Schematic overview of regenerative orthopedics Image created by the authors using Microsoft PowerPoint (Microsoft Corporation, Redmond, Washington, United States).

Although these developments have occurred, several important uncertainties and translation barriers remain. Heterogeneity in biologic preparations, the absence of standardized protocols, and inadequate mechanistic insights into the converging forces among immune, mechanical, and molecular microenvironments continue to lead to inconsistent regenerative outcomes. The reconstruction of the complex osteochondral interface, the stable and permanent integration of engineered constructs, and the enhancement of vascularization and innervation remain significant scientific challenges. Furthermore, other emerging modalities, including exosomes, nanomaterials, and bioprinted tissues, face challenges related to manufacturing scalability, regulatory approval, and cost-effectiveness that continue to hinder widespread clinical use. The gaps suggest we need a more detailed, well-planned synthesis of modern innovations in musculoskeletal tissue biology and regenerative orthopedic approaches. Recent studies in these domains have reported improved osteogenic and chondrogenic outcomes, enhanced biomaterial integration, and better modulation of inflammatory pathways, although variability in study design and methodologies remains a limitation. Figure 1 illustrates that regenerative orthopedics integrates advances in musculoskeletal tissue biology, engineering technologies, and supportive platforms to address clinical burden and translational challenges.

Objectives of the review

This review aims to synthesize current knowledge on musculoskeletal tissue biology and evaluate recent advances in regenerative orthopedic strategies, including orthobiologics, stem cell therapies, biomaterials, nanotechnology, and bioprinting. It further aims to identify key translational challenges, highlight clinical relevance, and outline future directions for improving the reliability and applicability of regenerative approaches in orthopedic practice.

Methodology

The present narrative review was constructed based on an extensive literature search to capture recent progress in musculoskeletal tissue biology and regenerative orthopedic methods, as outlined in the conceptual framework in the introduction. PubMed, Scopus, and Web of Science were used to retrieve peer-reviewed articles published primarily from 2020 to 2025 using keywords related to bone repair, cartilage repair, orthobiologics, stem cell therapies, biomaterials, nanotechnology, exosomes, mechanobiology, and 3D bioprinting. The literature search was conducted up to December 2025. Earlier foundational studies were also included where necessary to provide context for emerging concepts in tissue biology and regenerative approaches.

The eligible studies included basic science research, translational studies, preclinical models, and clinical applications directly related to biological or engineered strategies for bone and cartilage restoration, while non-orthopedic or methodologically weak studies were excluded. Inclusion criteria comprised peer-reviewed studies with clearly defined experimental or clinical methodologies, while studies lacking methodological transparency or clearly described outcome measures were excluded. Where available, studies describing orthobiologic preparation protocols, cell sourcing strategies, scaffold fabrication techniques, and exosome isolation methods were prioritized to improve reproducibility and translational relevance. As this is a narrative review, study selection was based on relevance to the topic rather than a formal systematic process, which may introduce potential selection bias. The collected data were synthesized descriptively to highlight mechanistic insights, therapeutic developments, scaffold technologies, orthobiologic effectiveness, and key translational challenges, providing a structured synthesis of current scientific advancements and existing gaps in regenerative orthopedics.

## Review

Advances in bone biology and mechanobiology

Current developments in skeletal biology now view skeletal regeneration as an extremely complex process in which osteoblasts, osteoclasts, osteocytes, endothelial cells, and immune mediators interact in a highly regulated system [22]. The balanced remodeling is regulated by the osteoblast-osteoclast coupling via the RANK, RANK ligand, osteoprotegerin, and macrophage colony-stimulating factor pathways and is mediated by the osteocytes as the mechanosensing cells which change mechanical stimuli into biochemical signals necessary to guide the repair [23]. The vascular compartment is also equally important, since angiogenic and osteogenic coupling mediate the recruitment of progenitor cells, nutrient transport, and mineralization, which become the biological basis of effective bone regeneration [12]. The immune-bone interactions have also been given a rising focus, in which the polarization of macrophages, cytokine gradients, and T-cell signaling display context-dependent influences on osteogenesis and remodeling [24].

**Figure 2:**
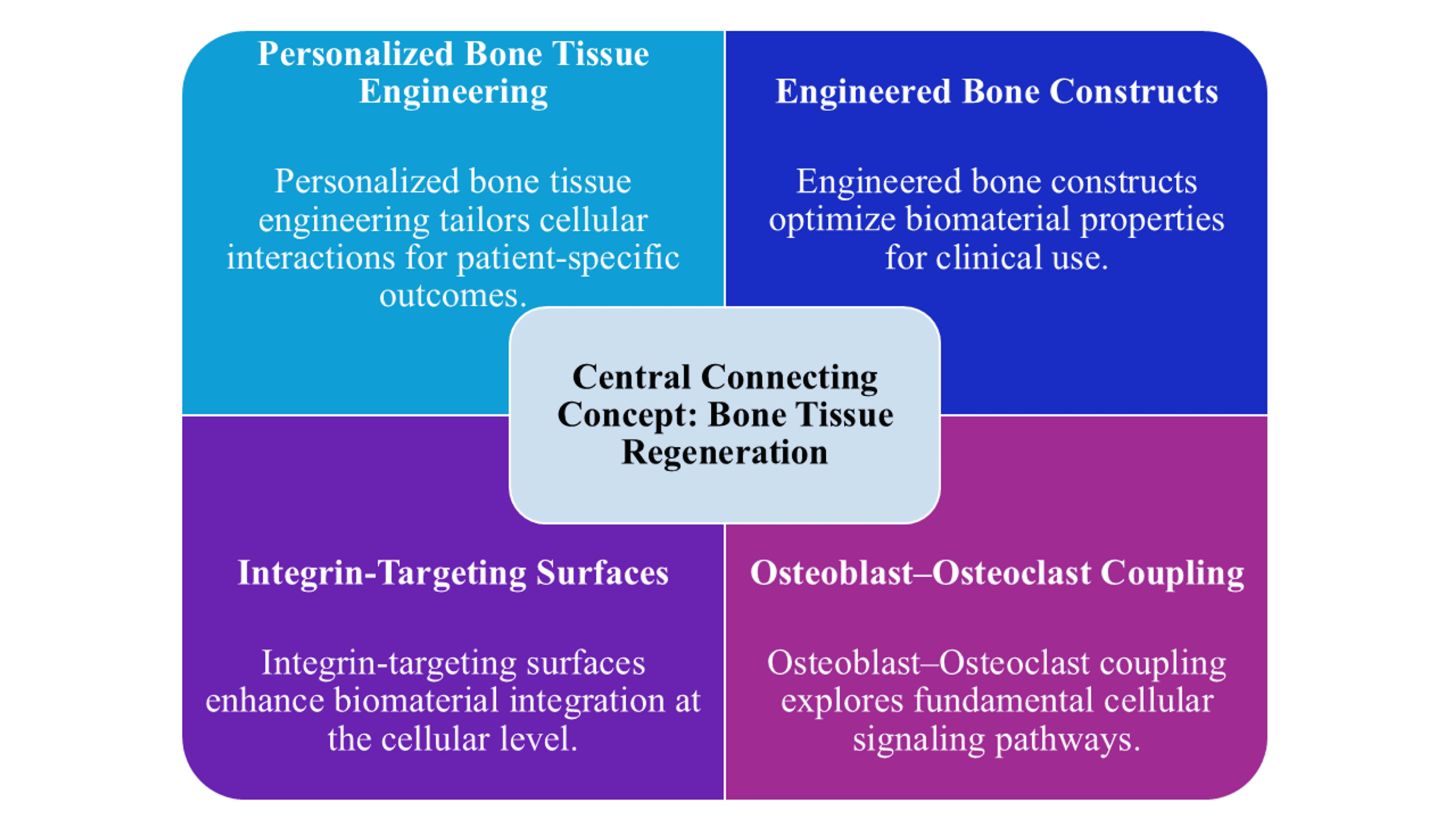
Mechanobiological and engineering drivers of bone regeneration Image created by the authors using Microsoft PowerPoint (Microsoft Corporation, Redmond, Washington, United States).

These mechanical understandings have played a major role in scaffold and biomaterials design [25]. Mechanotransduction pathways, such as Yes-associated protein, transcriptional coactivator with PDZ-binding motif, Wnt and beta-catenin signaling, and integrin-mediated pathways, give information on the engineering of constructs with optimized stiffness, porosity, and surface topography to facilitate osteogenic differentiation and cellular organization [14]. The mechanical competence of engineered tissues before implantation is now increasingly being improved using dynamic culture systems and preconditioning bioreactors [26]. Moreover, patient-specific (e.g., aging, metabolic disease, chronic inflammation, oxidative stress, etc.) factors have been identified as significant predictors of potential regeneration and support the idea of biologically informed and personalized regenerative bone tissue engineering [10]. Figure 2 shows that bone regeneration is governed by the integration of cellular coupling, mechanobiological signaling, biomaterial design, and patient-specific tissue engineering strategies.

Innovations in the cartilage microenvironment understanding

Regeneration of cartilages is also a significant problem because of the avascular character of the tissue, the zonal heterogeneity, and the low cellular turnover [27]. The most recent developments expanded the knowledge on the functional relevance of the specific zonal architecture of cartilage that is different in chondrocyte phenotype, collagen fiber orientation, and extracellular matrix composition [28]. This mechanical resilience and load-bearing capacity occur through this structural specialization, which has been the direct target of reconstruction in contemporary regenerative approaches [14]. The breakdown of the extracellular matrix, which is abundant in type II collagen, aggrecan, and glycosaminoglycans, disturbs osmotic equilibrium and disrupts chondrocyte homeostasis, hastening cartilage degeneration in degenerative diseases [29].

Molecular investigations have clarified mechanisms that control chondrogenesis and degeneration, such as transforming growth factor beta and bone morphogenetic protein signaling, which enhance the production of the matrix, and Wnt and beta-catenin signaling, which suppress hypertrophy and cartilage turnover [30]. These pathways dynamically interplay with the action of inflammatory mediators, including interleukin 1 beta, tumor necrosis factor alpha, and matrix metalloproteinases, which mediate the process of catabolic reactions in osteoarthritis and post-traumatic conditions [12]. Newer cartilage-on-chip and high-resolution three-dimensional co-culture systems now permit a high level of biomechanical loading, synovial-chondrocyte interactions, and disease-relevant inflammatory conditions to be modeled [31]. Physiologically relevant tools used to assess scaffold designs, orthobiologic therapies, and mechanotransductive cues are available with these systems, speeding the translation of cartilage regeneration technologies into more predictable clinical results [32]. These biological insights have guided the development of orthobiologic strategies aimed at enhancing the local regenerative environment.

Orthobiologics in regenerative orthopedics

The use of orthobiologics has become an important part of modern-day regenerative approaches that take advantage of the bioactive properties of autologous populations of cells, growth factors, and cytokine-containing preparations to regulate inflammation and facilitate tissue repair [33]. Platelet-rich plasma contains growth factors such as platelet-derived growth factor, transforming growth factor beta, vascular endothelial growth factor, and insulin-like growth factor 1, which contribute to matrix production, angiogenesis, as well as progenitor cell recruitment [12]. Forms of platelet-rich plasma and injectable platelet-rich fibrin vary in leukocyte concentration and release dynamics, which add to clinical responses in all musculoskeletal applications [34]. Marrow-derived cellular products and bone marrow aspirate concentrate are a heterogeneous combination of mesenchymal stromal cells, hematopoietic cells, and signaling molecules that synergize to produce osteogenesis, chondrogenesis, and immunomodulation [23].

The ability of orthobiologics to enhance fracture healing, cartilage repair procedures, as well as inflammation reduction in early osteoarthritis is clinically proven [35]. Mechanistically, these treatments enable transition of microenvironment to anti-inflammatory, stimulation of M2 macrophage polarization, angiogenic signaling, and anabolic signaling that is a core of tissue remodeling [14]. Nevertheless, the biological composition remains heterogeneous, commercial preparation procedures have not been standardized, and no large-scale randomized trials have been conducted to bring conclusive findings on efficacy [36]. New directions are focused on precision orthobiologics and preparations that are specific to patient biology, disease progression, and tissue regenerative requirements [24]. With a better grasp of mechanistic concepts, increasingly uniform techniques of processing, orthobiologics are likely to have an ever-increasing role in supplementing engineered constructs and cell-based methods in integrated regenerative orthopedic care [10]. Reported clinical outcomes include improved fracture healing rates, reduced pain scores in osteoarthritis, and enhanced functional recovery, although variability in preparation methods continues to limit reproducibility across studies [35,36]. Building on these approaches, stem cell-based therapies provide a more direct regenerative strategy through cellular differentiation and paracrine signaling. Table 1 shows that orthobiologic therapies enhance musculoskeletal repair through diverse bioactive mechanisms but are limited by variability and lack of standardization.

**Table 1:** Orthobiologics in regenerative orthopedics: composition, mechanisms, and clinical relevance PRP: platelet-rich plasma; i-PRF: injectable platelet-rich fibrin; PDGF: platelet-derived growth factor; TGF-β: transforming growth factor beta; VEGF: vascular endothelial growth factor; IGF-1: insulin-like growth factor-1; BMAC: bone marrow aspirate concentrate

Orthobiologic approach	Primary bioactive components	Key mechanisms of action	Clinical utility	Key limitations	Reference
PRP	PDGF, TGF-β, VEGF, IGF-1	Enhances extracellular matrix synthesis, promotes angiogenesis, recruits progenitor cells, and modulates inflammation	Fracture healing, cartilage repair, and early osteoarthritis	Variability in preparation methods and leukocyte concentration leads to inconsistent outcomes	Zhu et al. [13]
i-PRF	Platelets, fibrin matrix, leukocytes, cytokines	Sustained release of growth factors, immunomodulation, pro-angiogenic signaling	Cartilage repair, joint preservation	Limited standardization and long-term clinical evidence	Costa et al. [7]
BMAC	Mesenchymal stromal cells, hematopoietic cells, and cytokines	Osteogenesis, chondrogenesis, immunomodulation, M2 macrophage polarization	Nonunions, bone defects, cartilage regeneration	Cellular heterogeneity and donor-dependent variability	Lana et al. [8]
Marrow-derived cellular products	Mixed progenitor cells and bioactive signaling molecules	Synergistic anabolic signaling and regulation of the inflammatory microenvironment	Fracture healing and osteochondral repair	Lack of standardized processing protocols	Costa et al. [7]
Precision orthobiologics (emerging)	Patient-specific biologic formulations	Targeted immunomodulation and tissue-specific regenerative signaling	Personalized regenerative orthopedic therapies	Early developmental stage, regulatory and cost challenges	Navani et al. [16]

Stem cell-based therapies

Regenerative orthopedics has developed to be stem cell-based due to their capacity to regulate inflammation, stimulate matrix formation, and develop into either osteogenic or chondrogenic lineages [37]. The most studied obtained mesenchymal stromal cells are those of the bone marrow, adipose tissue, synovium, and periosteum, each with a specific biological benefit in its connection to the tissue of origin [23]. Mesenchymal stromal cells (obtained post-bone marrow) have good osteogenic capacity and have been effectively used in nonunions and complex fractures, but adipose-derived stem cells include a more reliable and easily available source with strong immunomodulatory and chondrogenic capabilities [38]. Synovium-derived mesenchymal stromal cells are more effective as chondrogenic cells and, therefore, are more appropriate in repairing the articular cartilage, whereas periosteal mesenchymal stromal cells are naturally committed to skeletal lineage and, therefore, are capable of bone tissue engineering [12].

In addition to their differentiation capabilities, mesenchymal stromal cells have strong paracrine activities due to the release of cytokines, growth factors, and extracellular vesicles that regulate local immune responses, promote angiogenesis, and trigger the activity of resident progenitor cells [24]. The pathways that control chondrogenesis include sex determining region Y box 9 and transforming growth factor beta-mediated pathways, and osteogenesis pathways require runt-related transcription factor 2, bone morphogenetic protein signaling, and integrin matrix signal transduction pathways [39]. These mechanistic understandings guide more and more the creation of specifically targeted cell-based therapy customized to tissues and stages of injury [10]. The mesenchymal stroma cell therapies have shown promising results in cartilage defects, early osteoarthritis, avascular necrosis, and bone regeneration, but the results of the treatment remain inconsistent [40]. The biggest problem is that the mesenchymal stromal cell preparations are heterogeneous in nature, since variations in the age of the donor, the tissue, isolation procedures, and expansion conditions can greatly affect therapeutic potency [26]. The legal limitations on cell manipulation and production also complicate the translation [30]. The restrictions have driven the focus towards cell-free approaches, specifically mesenchymal stromal cell-derived extracellular vesicles, which could present less risky and more standardized options [14]. Clinical studies have demonstrated improvements in cartilage repair and early osteoarthritis symptoms, although effect sizes remain inconsistent due to heterogeneity in cell sources and protocols [40]. However, stem cell-based therapies have continued to form the foundation of regenerative orthopedics, and, through continued optimization of delivery systems, dose, and patient selection, they are likely to enhance both their predictability and clinical effectiveness [22].

Biomaterials and scaffolds for bone and cartilage regeneration

Musculoskeletal regeneration has found biomaterial-based scaffolds essential as the framework to provide mechanical support, guide cellular responses, and deliver bioactive cues in a controlled manner [17]. The natural polymers like collagen, gelatin, and hyaluronic acid have high biocompatibility and biological recognition, which enables the adhesion of cells and enhances the deposition of the matrices [9]. In particular, hydrogel-based hydrogels comprised of hyaluronic acid have the potential to recapitulate the hydrated state of native cartilage and have demonstrated potential in promoting chondrogenesis and early cartilage remodeling [28]. Artificial polymers such as polycaprolactone and poly(lactic-co-glycolic acid) can provide adjustable degradation and mechanical strength that is effective in the reconstruction of bone defects [35]. These scaffolds in combination with ceramic materials like hydroxyapatite or beta tricalcium phosphate exhibit a higher osteoconductivity and replicate the composite structure of mineralized bone [14]. These hybrid materials facilitate healthy osteointegration, vascular tissue penetration, and mechanical stability, which are required to achieve long-term skeletal repair [6]. Advances in material design at the microscale have further evolved into nanoscale engineering approaches that enable more precise control of cell-material interactions.

Recent developments are concerned with the design of smart biomaterials that can dynamically respond to biological and mechanical stimuli [31]. Mechanoresponsive scaffolds are biomaterials that change their mechanical properties or structure in response to load, and these provide instructions to differentiate stem cells and facilitate the formation of matrices [22]. Temporal delivery of growth factors or chemotactic agents, which is necessary to achieve tissue repair successfully, can be achieved through controlled-release platforms incorporated into the scaffolds [11]. On the microscale and nanoscale, surface patterning, peptide functionalization, and topographical modulation optimize integrin-mediated signal transduction and cellular adhesion, growth, and commitment to a lineage [37]. Multilayered and gradient scaffolds used in cartilage regeneration aim to mimic the zonal structure of the native tissue using different mechanical stiffness, collagen direction, and biochemical constituents of the layers [4]. Osteochondral scaffolds combine subchondral mineral phases and cartilage mimetic surfaces to re-establish biomechanical continuity across the interface [30]. Regardless of these developments, there are still problems such as a lack of long-term durability, partial incorporation with host tissue, and vascularization of composite constructs [25]. Further developments in the science of biomaterials, which are combined with orthobiologic and mechanobiological understanding, are likely to lead to more predictable acquisition of bone and cartilage [19]. Table 2 shows that scaffold design and material composition critically influence mechanical support, biological performance, and integration in bone and cartilage regeneration.

**Table 2:** Biomaterial-based scaffolds for bone and cartilage regeneration: composition, function, and challenges

Scaffold type	Material composition	Primary functional role	Target tissue/application	Key limitations	Reference
Natural polymer scaffolds	Collagen, gelatin, hyaluronic acid	High biocompatibility, biological recognition, enhanced cell adhesion and matrix deposition	Cartilage regeneration, soft tissue repair	Limited mechanical strength and long-term durability	Liang et al. [9]
Hyaluronic acid-based hydrogels	Hyaluronic acid hydrogels	Recapitulation of hydrated cartilage environment, promotion of chondrogenesis and early matrix remodeling	Articular cartilage repair	Mechanical weakness in load-bearing environments	Vaish and Vaishya [28]
Synthetic polymer scaffolds	Polycaprolactone, poly(lactic-co-glycolic acid)	Tunable degradation rates and mechanical strength	Bone defect reconstruction	Limited bioactivity without modification	Georgeanu et al. [35]
Hybrid polymer-ceramic scaffolds	Synthetic polymers combined with hydroxyapatite or β-tricalcium phosphate	Enhanced osteoconductivity, support for vascular infiltration, and osteointegration	Bone regeneration and skeletal repair	Complexity of fabrication and integration	Łuczak et al. [6]
Smart and mechanoresponsive biomaterials	Stimuli-responsive polymers and composites	Dynamic adaptation to mechanical loading, guidance of stem cell differentiation	Bone and cartilage tissue engineering	Early-stage development and translational challenges	Eskandar [22]
Controlled-release scaffolds	Growth factor- or chemokine-loaded biomaterials	Temporal delivery of bioactive signals to coordinate tissue repair	Bone and cartilage regeneration	Difficulty achieving precise release kinetics	Winkler et al. [11]
Multilayered and gradient scaffolds	Zonal variations in stiffness, collagen orientation, and biochemical cues	Mimicry of native cartilage zonal architecture	Cartilage regeneration	Incomplete long-term integration	Frączek et al. [4]
Osteochondral scaffolds	Mineralized subchondral phase with cartilage-mimetic surface	Restoration of biomechanical continuity across the osteochondral interface	Osteochondral defect repair	Insufficient vascularization and interface stability	Zhang et al. [30]

Nanotechnology-based approaches

The new approach to musculoskeletal regeneration brought about by nanotechnology is the ability to regulate the interactions of cell materials precisely and deliver therapy specifically where it is needed [41]. Electrospun nanofibrous scaffolds are similar to extracellular matrix architecture, which offers aligned or disordered fiber networks that direct cell morphology, movement, and differentiation [12]. These nanoscaffolds enhance chondrogenic and osteogenic functions through the regulation of cytoskeletal tension and triggering of important mechanotransduction signaling [29]. Nanoparticles are strong vectors of growth factors, nucleic acids, and anti-inflammatory agents, permitting a regulated and localized release and reducing systemic exposure [42]. Not only do bioactive nanoparticles like calcium phosphate, bioactive glass, gold, and graphene derivatives deliver therapeutic payloads, but they also directly regulate regeneration by facilitating mineralization, osteogenesis, and oxidative stress [18]. Their small size makes it easy to penetrate thick tissues and enhance contact with cellular and extracellular materials [33].

Nanostructured scaffolds that include nanotubes, nanopores, or patterned nanosurfaces facilitate protein adsorption, integrin clustering, and early cell adhesion, resulting in a more robust matrix production [43]. Nanomaterials and multifunctional nanoproducts can be engineered to possess both regenerative and diagnostic capabilities, enabling real-time imaging, scaffold tracking, and responsiveness to external stimuli such as magnetic fields or light [25]. Combining nanotechnology with new modalities such as three-dimensional bioprinting, stem cell therapies, and intelligent biomaterials is driving the formation of more complex and biologically responsive constructs [44].

Bioprinting and advanced tissue engineering

The recent advancements in musculoskeletal regeneration have centered on the use of tissue engineering and three-dimensional bioprinting to fabricate constructs that recapitulate the architecture, cellular structure, and mechanical characteristics of normal bone and cartilage [45]. The first scaffold-based systems offered structural support at an early stage but were not as sophisticated as functional regeneration needs [12]. Recent advances in biomaterials, cell biology, and additive manufacturing have overcome these constraints in the context of providing more precise spatial control of cells, matrix components, and biochemical cues [30]. However, the core of these methods is the fabrication of bioinks, which are made of hydrogels, extracellular matrix-derived components, and lineage-specific cells like mesenchymal stromal cells, chondrocytes, and osteoblasts [46]. These bioinks have tunable mechanical stiffness and biochemical profiles and can be used to sustain cell viability, differentiation, and extracellular matrix deposition [22]. The three-dimensional bioprinting technologies currently allow depositing in layers each type of material and cell and making complex tissue interfaces like the osteochondral unit [47]. Gradient scaffolds based on zonal cartilage architecture or mineralized bone transitions can improve functional integration and permit physiologically relevant load distribution [16]. Structural competence of bioprinted constructs has been enhanced using reinforcement methods, which include the addition of ceramic particles, polymeric meshes, or composite phases, which overcome the mechanical weakness issue associated with hydrogel-dominated systems [48].

**Figure 3:**
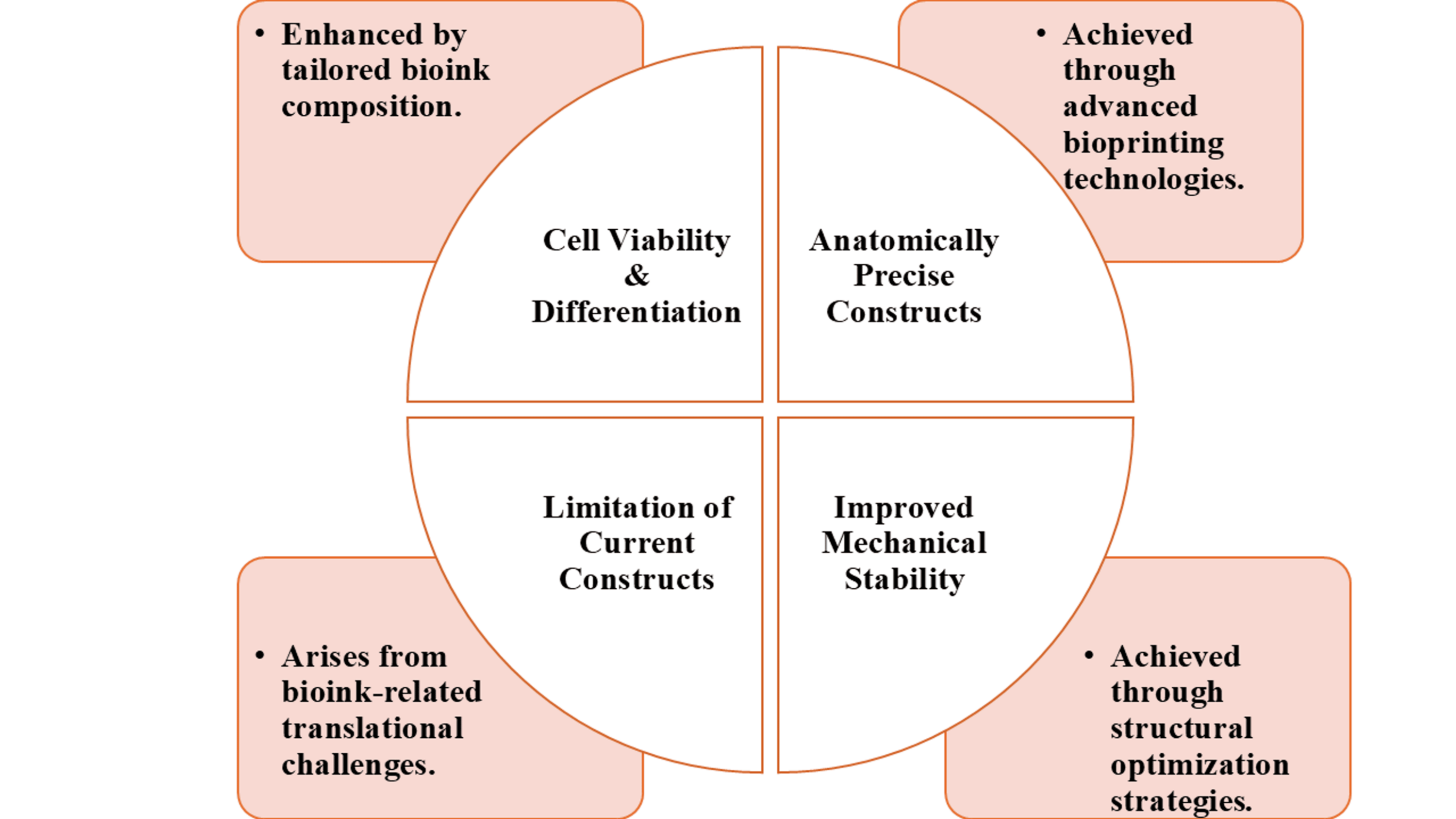
Tissue engineering and 3D bioprinting in musculoskeletal regeneration Image created by the authors using Microsoft PowerPoint (Microsoft Corporation, Redmond, Washington, United States).

However, a number of difficulties still exist [11]. The biggest obstacle is vascularization, where engineered tissue should be rapidly perfused to maintain cell viability and for the maintenance of regenerative mechanisms [35]. Solutions, including incorporating endothelial cells, printing microchannels, or incorporating angiogenic factors, are under active investigation to solve this requirement [49]. There are also no final words on mechanical durability and long-term interface integration, especially on constructs that are supposed to undergo high load conditions, like on large bone defects or weight-bearing cartilage [28]. Future developments in immunomodulatory biomaterials, mechanically adaptive matrices, and dynamic culture systems provide potential opportunities to enhance construct maturation and in vivo performance [37]. Combined, tissue engineering and three-dimensional bioprinting are emerging technologies with significant growth potential to produce anatomically viable and biologically functional musculoskeletal regeneration [50]. Despite this progress, most of these technologies remain at the preclinical or early clinical stage, and large-scale randomized clinical evidence is still limited. Figure 3 shows that effective musculoskeletal regeneration through 3D bioprinting depends on balancing bioink design, anatomical precision, and mechanical stability despite existing translational limitations.

Exosome and cell-free therapies

Cell-free therapies and especially exosome-based therapies have become formidable alternatives to cell transplantation, through the exploitation of the paracrine-based regeneration via stem cell-mediated regeneration [17]. Exosomes, which are derived from mesenchymal stromal cells or other progenitor cells, wrap microRNAs, proteins, and lipids that regulate inflammation, angiogenesis, and matrix synthesis [21]. Their ability to penetrate tissues effectively due to their nanoscale size and membrane stability and to avoid the degradation of bioactive cargo allows cells in an injury microenvironment to be regulated precisely, thereby suppressing immune responses [29]. Exosomes improve osteogenic differentiation by promoting bone repair by delivering osteoinductive microRNAs and activating bone morphogenetic protein and Wnt signaling pathways [13]. They induce angiogenesis, which is a crucial process in effective bone regeneration through activating endothelial migration and proliferation [25]. Exosomes play an important role in cartilage repair by inhibiting catabolic signaling through the inhibition of inflammatory mediators and matrix-degrading enzymes and stimulating the proliferation of chondrocytes, extracellular matrix synthesis, and redifferentiation [19]. Their immunomodulatory properties, as well as the facilitation of anti-inflammatory macrophage phenotypes, also contribute to the regenerative intra-articular environment [30].

Exosomes have clear benefits over cell therapy, such as reduced immunogenicity, storage capability, scalability, and predictable safety profiles [12]. These advantages make exosome-based therapies attractive from a translational perspective, particularly where standardization, storage, and safety are major barriers to routine cell-based therapy. Nevertheless, there are still serious obstacles, especially in the standardization of isolation techniques, the description of the cargo content, the maximization of dosing, and the realization of consistent therapeutic efficacy [26]. As a result, current evidence supports exosomes as a promising adjunctive platform rather than a fully established standalone clinical solution. The regulatory routes of extracellular vesicle-based products remain under development, and long-term safety and biodistribution need to be further explored [14]. These obstacles notwithstanding, exosomes are a promising new generation of orthobiologic treatment that can provide specific, cell-free control over musculoskeletal regeneration [18].

Rehabilitation-integrated regenerative strategies

Regenerative rehabilitation combines the concepts of biomechanics, tissue engineering, and orthobiologics to promote tissue repair using controlled mechanical stimulation [42]. Mechanical loading is a key determinant of musculoskeletal homeostasis affecting stem cell differentiation, extracellular matrix alignment, and angiogenesis via integrin signaling, ion channel activation, and YAP and transcriptional coactivator with PDZ binding motive signaling pathways [17]. It has been understood that mechanical cues hold biological significance and, in that connection, the current rehabilitation paradigm would focus on aligning the physical therapy regimens with the biological stages of tissue healing [8]. In bone regeneration, early controlled loading enhances osteogenic differentiation, enhances angiogenesis, and enhances callus formation, and excessive or too early loading is associated with construct failure [33]. Tailored low-impact mechanical stimulation can aid in the recovery of chondrocyte function, fibril organization, and deposition of proteoglycan-rich matrix in cartilage repair [21]. Individualized rehabilitation interventions by using gait measurement, sensor-based monitoring, and adaptive load control allow clinicians to tailor treatment intensity in accordance with the patient's unique healing patterns [49].

Biologic therapies are being combined with emerging technologies, such as robot exoskeletons, mechanotherapy machines, and electronic monitoring systems, to maximize mechanical environment-directed regeneration [12]. Early evidence suggests a synergistic effect when mesenchymal stromal cell therapies, platelet-rich plasma injections, or engineered constructs are combined with targeted mechanical stimulation [28]. This integrated approach is clinically relevant because biologic repair alone may not ensure durable functional recovery unless the postoperative mechanical environment is also optimized. As regenerative rehabilitation continues to develop, it is set to be instrumental in mediating the biological repair to lasting functional restoration [36].

Clinical translation and challenges

Despite significant technological progress, translation of these approaches into routine clinical practice remains challenging. Regardless of the significant advances, regenerative orthopedic treatments have a number of translation and clinical obstacles [32]. These challenges indicate that therapeutic promise has progressed faster than clinical standardization, which partly explains the variability in published outcomes. Biological variability, differences in processing methods, and patient-specific factors contribute to inconsistent and sometimes unpredictable outcomes [27]. Standardization is still a pressing requirement, especially in the case of orthobiologics and exosome-based therapeutics, where the method of preparation has an immediate impact on the biological activity [41]. This is particularly important because differences in preparation protocols may influence cell composition, growth factor release, cargo profile, and ultimately therapeutic performance. The complexities of regulatory requirements relating to cell manipulation, biomaterial engineering, and nanotechnology also slow the uptake of clinical use [38]. Economic factors, such as cost-effectiveness studies, long-term value studies, and reimbursement systems, have also not been well developed to enable their broad application in clinical practice [44]. Regenerative methods are increasingly being applied in fractures, osteochondral repair, focal cartilage lesions, early osteoarthritis, and biologic augmentation of soft tissue procedures [23]. These applications are particularly relevant in conditions such as early osteoarthritis, nonunion fractures, and focal cartilage defects where conventional treatments may have limited long-term success. Early clinical studies have reported promising outcomes with bone marrow aspirate concentrate in nonunions, platelet-rich plasma or mesenchymal stromal cells in early osteoarthritis, and scaffold-assisted cartilage repair in focal defects [31]. Restoration of structure and function across complex tissue interfaces is seen in engineered osteochondral constructs, which are still in early clinical testing [39].

Precision medicine strategies based on genomic, proteomic, and biomechanical profiling may improve future clinical integration by helping match regenerative therapies to individual patient characteristics [45]. The development of manufacturing, quality control, and long-term monitoring of outcomes will be crucial towards establishing the reliability and safety of next-generation products [28]. Regenerative orthopedics has strong potential to become a major component of musculoskeletal care, but broader adoption will depend on overcoming translational barriers and strengthening the quality of clinical evidence [34]. Table 3 shows that successful clinical translation of regenerative orthopedics depends on overcoming biological variability, standardization, regulatory, and economic barriers while leveraging emerging clinical evidence.

**Table 3:** Translational challenges and emerging clinical applications in regenerative orthopedics PRP: platelet-rich plasma; MSCs: mesenchymal stem cells; BMAC: bone marrow aspirate concentrate

Category	Key issues or developments	Impact on clinical translation	Examples/current status	References
Biological and patient variability	Variability in biologic products, processing techniques, and patient-specific factors	Leads to inconsistent and unpredictable clinical outcomes	Particularly evident in orthobiologics and cell-based therapies	De Pace et al. [27]
Lack of standardization	Non-uniform preparation and characterization of biologics and exosome-based therapies	Directly affects biological activity and reproducibility	Major limitation for orthobiologics and extracellular vesicle therapies	Tuan et al. [41]
Regulatory challenges	Complex regulations governing cell manipulation, biomaterials, and nanotechnology	Slows clinical adoption and large-scale translation	Varies across regions and product classes	Hung et al. [38]
Economic and reimbursement barriers	Limited cost-effectiveness data, long-term value assessment, and reimbursement frameworks	Restricts widespread clinical implementation	Particularly relevant for advanced biologics and engineered constructs	Yang et al. [44]
Expanding clinical applications	Increasing use of regenerative therapies in routine orthopedic practice	Demonstrates growing translational momentum	Fractures, nonunions, osteochondral defects, and early osteoarthritis	Hernigou et al. [23]
Evidence from clinical trials	Promising outcomes reported for biologics and scaffold-assisted repair	Supports feasibility but requires long-term validation	BMAC for nonunions, PRP or MSCs for early osteoarthritis	Jones et al. [31]
Engineered osteochondral constructs	Early-stage clinical evaluation of interface-restoring constructs	Potential to restore structure and function across complex tissues	Still limited to early clinical testing	Lana et al. [39]
Precision medicine approaches	Genomic, proteomic, and biomechanical profiling for therapy customization	Enables patient-specific regenerative strategies	Emerging direction for future clinical integration	Wang et al. [45]
Manufacturing and quality control	Need for scalable manufacturing, standardized quality control, and long-term outcome monitoring.	Essential for safety, reliability, and regulatory approval	Critical for next-generation regenerative products	Vaish and Vaishya [28]
Future outlook	Progressive removal of translational barriers and expansion of evidence-based approaches are essential for effective clinical implementation	Positions regenerative orthopedics as a core component of musculoskeletal care	Transition from experimental to routine clinical use	Santoro et al. [34]

Limitations and future directions

Regenerative orthopedic technologies still face significant limitations that reduce consistency, reproducibility, and clinical reliability. Considerable heterogeneity in study design, biologic preparation methods, and outcome reporting makes comparison across studies difficult and limits the development of standardized treatment protocols. For emerging modalities such as exosomes, smart biomaterials, and 3D-bioprinted constructs, long-term safety and efficacy data remain limited. Additional challenges include reproducing the structural and biochemical complexity of the osteochondral interface and achieving durable vascularization and mechanical integration. Differences in donor biology and processing methods further contribute to variation in orthobiologic potency, while regulatory and manufacturing constraints highlight the need for strict quality control and reproducibility standards.

Future development should move toward more precise regenerative strategies that integrate molecular profiling, biomechanics, and AI-assisted predictive modeling to match interventions to individual patient needs. Intelligent and immunomodulatory biomaterials, self-healing scaffolds, and dynamic matrices may improve tissue responsiveness and functional integration. Safer and more scalable alternatives to cell-based therapies may emerge through engineered exosomes and other cell-free platforms, while advances in vascularization, innervation, and organ-on-chip modeling may improve translation from preclinical studies to clinical application. Stronger regulatory frameworks, standardized manufacturing pathways, and more multicenter clinical trials will be necessary to validate these technologies and support broader clinical adoption.

## Conclusions

Regenerative orthopedics is undergoing substantial progress driven by advances in musculoskeletal tissue biology, biomaterials engineering, and cellular and molecular therapeutics. Greater insight into the biological and biomechanical factors underlying bone and cartilage repair has been enabled by the development of orthobiologics, stem cell-based interventions, nanotechnology platforms, and 3D-bioprinted scaffolds, which, in turn, more realistically replicate native tissue structure and function. Although these innovations show considerable promise, their clinical impact remains limited by inconsistent biologic preparations, insufficient long-term outcome data, difficulties in achieving robust osteochondral integration, and evolving regulatory frameworks. Overcoming these limitations will require greater standardization of biologic processing, rigorous long-term clinical evaluation, and precision medicine models that align regenerative approaches with patient-specific biological and biomechanical characteristics. Continued interdisciplinary collaboration will be necessary to refine emerging technologies, improve translational efficiency, and ensure safety, reproducibility, and clinical practicality. With continued scientific validation and clinical refinement, regenerative orthopedics may provide more reliable, durable, and personalized treatment options for musculoskeletal disease and improve future standards of care.
